# Multifocal ectopic purkinje-related premature contractions and related cardiomyopathy

**DOI:** 10.3389/fcvm.2023.1179018

**Published:** 2023-08-04

**Authors:** Kirstine Calloe, Helena B. D. Magnusson, Dorte Launholt Lildballe, Morten Krogh Christiansen, Henrik Kjærulf Jensen

**Affiliations:** ^1^ Department of Veterinary and Animal Sciences, University of Copenhagen, Frederiksberg, Denmark; ^2^ Department of Molecular Medicine, Aarhus University Hospital, Aarhus, Denmark; ^3^ Department of Cardiology, Aarhus University Hospital, Aarhus, Denmark; ^4^ Department of Clinical Medicine, Aarhus University, Aarhus, Denmark

**Keywords:** dilated cardiomyopathy, premature ventricular contractions, MEPPC, *SCN5A*, arrhythmia

## Abstract

In the past 20 years, genetic variants in *SCN5A* encoding the cardiac voltage-gated sodium channel Na_v_1.5 have been linked to a range of inherited cardiac arrhythmias: variants resulting in loss-of-function of Na_v_1.5 have been linked to sick sinus syndrome, atrial stand still, atrial fibrillation (AF) impaired pulse generation, progressive and non-progressive conduction defects, the Brugada Syndrome (BrS), and sudden cardiac death. *SCN5A* variants causing increased sodium current during the plateau phase of the cardiac action potential is associated with Long QT Syndrome type 3 (LQTS3), *Torsade de Pointes* ventricular tachycardia and SCD. Recently, gain-of-function variants have been linked to complex electrical phenotypes, such as the Multifocal Ectopic Purkinje-related Premature Contractions (MEPPC) syndrome. MEPPC is a rare condition characterized by a high burden of premature atrial contractions (PACs) and/or premature ventricular contractions (PVCs) often accompanied by dilated cardiomyopathy (DCM). MEPPC is inherited in an autosomal dominant fashion with an almost complete penetrance. The onset is often in childhood. The link between *SCN5A* variants, MEPPC and DCM is currently not well understood, but amino acid substitutions resulting in gain-of-function of Na_v_1.5 or introduction of gating pore currents potentially play an important role. DCM patients with a MEPPC phenotype respond relatively poorly to standard heart failure medical therapy and catheter ablation as the PVCs originate from all parts of the fascicular Purkinje fiber network. Class 1c sodium channel inhibitors, notably flecainide, have a remarkable positive effect on the ectopic burden and the associated cardiomyopathy. This highlights the importance of genetic screening of DCM patients to identify patients with *SCN5A* variants associated with MEPPC. Here we review the MEPPC phenotype, MEPPC-*SCN5A* associated variants, and pathogenesis as well as treatment options.

## Introduction

The depolarization phase of the cardiac action potential is due to the activation of the voltage-gated sodium channel Na_v_1.5 encoded by the gene *SCN5A*.

Genetic variants in *SCN5A* causing a loss-of-function of Na_v_1.5 have been linked to sick sinus syndrome, atrial stand still, atrial fibrillation (AF), progressive and non-progressive cardiac conduction disease, the Brugada syndrome (BrS) and sudden cardiac death (SCD). *SCN5A* variants causing increased sodium current during the plateau phase of the cardiac action potential is associated with Long QT Syndrome type 3 (LQTS3), *Torsade de pointes* ventricular tachycardia and SCD. The different disease mechanisms are excellently reviewed by Wilde and Amin (1). These pathologies are often inherited in an autosomal dominant manner with incomplete penetrance. Furthermore, the manifestations of *SCN5A* variants are highly variably and the same genotype can be associated with multiple and overlapping phenotypes (2).

Recently, the Multifocal Ectopic Purkinje-related Premature Contractions (MEPPC) syndrome has been added to the list of *SCN5A* associated phenotypes. MEPPC is a rare, but likely underdiagnosed, syndrome in which the presence of multiple ectopic polymorphic ventricular complexes (PVCs) with relatively narrow QRS complexes is a central feature. MEPPC was first described by Laurent and coworkers in 2012 based on three families with the c.665G > A *SCN5A* variant resulting in the R222Q amino acid substitution in Na_v_1.5. At almost the same time, other reports on R222Q in families with complex arrhythmias and dilated cardiomyopathy (DCM) were published (3, 4). After these initial reports, additional *SCN5A* variants have been suggested to cause an MEPPC phenotype or an MEPPC-like phenotype.

Here, we review the MEPPC phenotype, MEPPC-*SCN5A* associated variants, and pathogenesis as well as management options.

## Clinical presentation and diagnostic criteria

**Figure 1:**
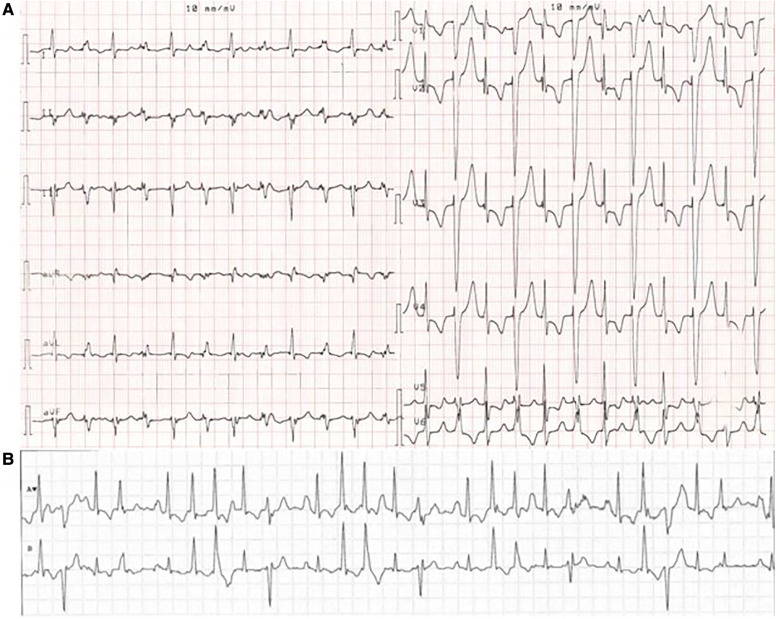
ECG from a MEPPC patient with the G213D variant. (**A**) ECG with PVCs as bigemini. (**B**) Telemetry of the patient showing a chaotic ECG with rare sinus beats interpolated between frequent PVCs, premature atrial complexes and premature junctional complexes. The figure is reprinted from International Journal of Cardiology, Calloe et al. (6) with permission from Elsevier.

MEPPC patients typically present with palpitations, dyspnea, syncope, or SCD (5). The age of onset varies, but the clinical symptoms are often found at a young age (6) or during childhood (5). The electrical phenotype is characterized by multiple premature ventricular complexes (PVCs) originating from the fascicular Purkinje system, including narrow complexes (high septal) and wide complexes (left or right bundle branch block patterns) morphologies. It is not uncommon for the patients to have more than 50.000 PVCs/24 h (5, 6). Often premature atrial complexes (PACs), AF and varying conduction abnormalities are concomitant (Figure 1). Occasionally, ventricular tachycardia (predominantly non-sustained) and associated syncope or SCD may be observed (5).

The diagnostic criteria for MEPPC have not been defined and often MEPPC-like syndromes are reported as complex arrhythmia and DCM. A central feature of MEPPC is the presence of multiple PVCs with relatively narrow QRS complexes or typical left/right branch block patterns indicating an origin from the fascicular Purkinje system, in which case a diagnosis of MEPPC should be suspected. The diagnosis may further be supported by the presence of a positive family history of PVCs and/or sudden cardiac death and a *SCN5A* gain-of-function variant. In contrast to other *SCN5A*-associated arrhythmia phenotypes, ST-segment morphology and QT interval during sinus beats appear to be normal.

For patients with the R222Q (5) variant or one of the two later reported variants G213D (6) and Q419P (7), the number of PVCs decline during exercise, likely due to overdrive suppression. Structural changes with dilation of the atria and ventricles as well as impairment of left ventricular systolic function is frequently found (3–5, 8) and left ventricular fibrosis has been observed in MEPPC patients with left ventricular dysfunction (9, 10).

MEPPC exhibits an autosomal dominant inheritance pattern with almost complete penetrance (3, 5, 6). There is an equal distribution of male and female MEPPC patients, but DCM and reduced left ventricular ejection fraction (LVEF) appear to be more frequently in males than in females. For patients with the R222Q variant almost twice as many males as females (9 vs. 5) presented with DCM (3–5). Of the 39 genotypic positive family members, all except one had cardiac arrhythmic ectopies (3–5). Calloe and coworkers identified 15 cases with the amino acid substitution G213D. DCM was found in three genotype positive male patients and in two male family members, where MEPPC was suspected but genetic testing not possible. DCM was not found in female carriers of this variant, with exception of a 14 year-old female with atrial tachycardia and an enlarged left atrium (6).

## Na_v_1.5 gating pore currents and MEPPC

Considering the central role of Na_v_1.5 in cardiac action potential generation and conduction, the association between pathogenic *SCN5A* variants and cardiac arrhythmia is not surprising. Variants in *SCN5A* is found in 1.7% of familiar cases of DCM (11) but the mechanistic link between Na_v_1.5 variants and DCM is still a subject of debate.

A recent systematic review by Peters and coworkers identified 18 DCM-associated Na_v_1.5 variants (12) and they concluded that the majority of DCM-associated variants were also associated with PVCs. Out of these 18 DCM-associated Na_v_1.5 variants, A204E (9), G213D (6, 13), R222Q (3–5, 14), R225P (15), Q419P (7), and L828F (16) have been reported as causing MEPPC or MEPPC-like syndromes (Tables 1, 2). M1851V (27) and I141P (26) are causing an atypical MEPPC-like phenotype where PVCs are induced by excise rather than being suppressed (Table 2). Other variants, like R814W (22–24) appear to evoke an MEPPC-like phenotype (Table 1), but are reported as causing “multifocal PVCs and DCM” in the literature.

**Table 1:** *SCN5A* variants associated with MEPPC. Minor allele frequency (MAF) based on (https://gnomad.broadinstitute.org/gene/ENSG00000183873?dataset = gnomad_r2_1).

MEPPC associated *SCN5A* variants (NM_198056)	MAF (gnomAD exomes/genomes) and number of patients in litterature	Phenotype	Electrophysiological characteristics of Na_v_1.5 with the amino acid substitution	Treatment
c.611C > A p.A204E (9)	0–0 1 patient	PVC, no info on the effect of exercise LV dilated, DCM	Activation at more negative voltages Increased window current	Hydroquinedine (9)
c.638G > A p.G213D (6, 13)	0–0 20 patients, 17 genotype positive j	PVCs disappear during exercise DCM Normal QTc,	Activation at more negative voltages Increased window current No late current (6) Gating pore current, inward current at negative potentials. In hiPSC derived the resting membrane potential depolarized with a high frequency action potentials (13)	Flecainde (6, 13)
c.665G > A p.R222Q (3–5, 14, 17–20)	0–0.00000657 (1/152,176 age above 50: 1) 73 patients, 59 genotype positive	PVCs disappear during exercise DCM Normal QTc,	Activation at more negative voltages Increased window current No late current (4) Gating pore current, outward current at depolarized potentials (4, 5, 14, 17)	Hydroquinedine (5) Lidocain (18) Quinedine (19) Flecainide(3)
c.674G > C p. R225P (15)	0–0 1 patient (fetal onset)	PVC DCM	Almost a 2-fold increase in peak current Small hyperpolarized shift in activation Late current Increased window current Gating pore currents, outward at depolarised potentials (21)	Amiodarone
c.1256A > C p.Q419P (7)	0–0 5 patients, 4 genotype positive	PVCs disappear during exercise Early onset AF No DCM Normal QTc,	Activation at more negative voltages Increased window current (7)	Flecainide (7)
c.2440C > T p.R814W (22–24)	0–0 6 patients, 4 genotype positive	Atrial flutter PVC Tachycardia Normal QTc DCM Suppressed by overdrive pacing, no info on the effect of exercise. Not reported as MEPPC.	Activation at more negative voltages Slower activation kinetics Increased window current (23) Gating pore currents, outward at depolarized potentials (21)	Quinidine/mexiletine (24)
c.2482C > T p.L828F (16)	0–0 4 patients, 2 genotype positive	PVC increase with emotional stress, no info on the effect of exercise DCM Small family and a KCNE1 variant is present at least in one patient but normal QTc	Increased peak currents Activation at more negative voltages Increased window current	Flecainide (16)

The number of patients described in the literature as well as number of confirmed genotype positive patients. The number of patients often exceeds genotype positive patients as not all patients agreed to genetic testing and in some cases it was not possible.

**Table 2:** SCN5A variants associated with complex arrhythmias and DCM. Minor allele frequency (MAF) based on (https://gnomad.broadinstitute.org/gene/ENSG00000183873?dataset = gnomad_r2_1).

Variants in *SCN5A* associated with complex arrhythmias and/or DCM (NM_198056)				
c.421A > G p.I141V (26)	0–0 16 patients, 20 genotype positive	Low PVC burden (polymorph, multifocal) PVCs induced by exercise Early onset AF Structurally normal hearts Reported as MEPPC	Negative shift in activation Increased window currents	Betablockers (26)
c.5551A > G p.M1851V (27)	0–0.00000657 (1/152,144; Age above 50:1) 8 patients, 8 genotype positive	Low PVC burden (polymorph, multifocal) PVCs induced by exercise Early onset AF Mild structural changes Normal QTc Reported as MEPPC	No shift in activation Depolarized shift in inactivation Window current increased Slow onset of inactivation	Betablockers (27)
c.589G > C p.D197H (28)	0–0 6 patients, 6 genotype positive	Atrial standstill Conduction disease Ventricular tachycardia DCM Some family members carries an *NEXN* variant	No data The D197H substitution may cause gating pore current (29)	No data
c.656G > A p.R219H (30, 31)	0–0.00000657 (1/152,116; Age above 50: unknown) 6 patients, 3 genotype positive	Bradycardia Tachycardia Conduction disorder PVC DCM	Properties of the sodium current not altered, no late current Proton gating pore current, inward H^+^ leak at negative potentials (30) In hiPSC derived cardiomyocytes the action potential duration was increased and the resting membrane potential was depolarized (31)	ACE-inhibition, diuretics and betablockers (30)
c.673C > T p.R225W (17, 32)	0.0000124 (3/242,066) – 0.0000788 (12/152 200; Age above 50:3; Age below 30: 2)	Conduction disorder Wide QRS complexes The phenotype is complicated by the presence of the loss-of-function W156X variant Tachycardia (32)	Reduced peak currents Activation at more positive voltage Inactivation released at less negative potentials (32) Increased window currents Gating pore current, outward current at depolarized potentials (17)	Betablockers (32)
c.3740C > T p.T1247I (33)	0.0000318 (8/251 380; Age above 50: 4) 0.00004501 (7/155,504) 19 patients	DCM Hypertrophic cardiomyopathy	No apparent phenotype Small left shift of activation Slow onset of inactivation (34) The T1247I substitution may cause gating pore current (29)	No data
c.3823G > A p.D1275N (18, 22, 35, 36)	0.00000796 (2/251 372; Age above 50: 1) 25 patients, 25 genotype positive	AF Conduction delay Normal QTc DCM	Reduced peak current Late current D1275N may cause gating pore current based on its position (29)	
c.4877G > A p.R1626H (37, 38)	0.0000398 (10/251 192; Age above 50: 7) 0.0000263 (4/152 024)	Lone AF (37) LQTS3 (38) VT and DCM (unpublished observation)	Reduced peak currents Inactivation is markedly slowed Late current (37) The R1626H substitution may cause a proton gating pore current based on its position (29)	

The number of patients described in the literature as well as number of confirmed genotype positive patients. The number of patients often exceeds genotype positive patients as not all patients agreed to genetic testing and in some cases it was not possible. LQTS3 variants that cause hyper excitable Purkinje fibers (25) are not included in the table.

**Figure 2:**
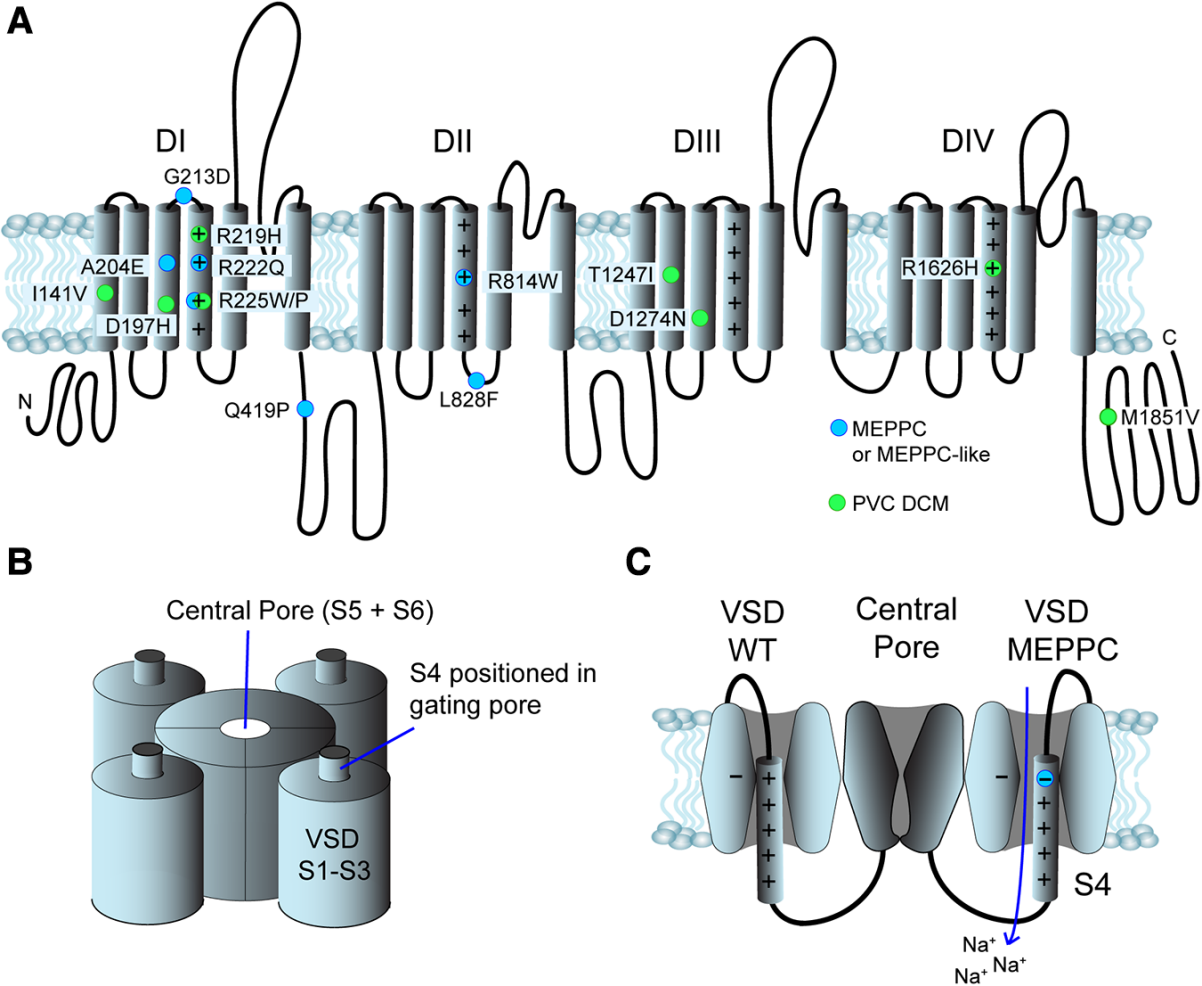
Structure and position of variants in the Na_v_1.5 channel. (**A**) Primary structure of the Na_v_1.5 channel. It consists of 4 domains (DI to DIV), each with 6 transmembrane segments (S1–S6). The positive residues in S4 are indicated by “+”. The N- and C- termini are found in the cytoplasm. Blue dots indicate the position of MEPPC variants and green dots indicate variants with complex arrhythmias and DCM. (**B**) The S5 and S6 from homologue domains form the central pore and the selectivity filter. S1–S4 form the voltage sensitive domains (VSD), S1–S3 form a gating pore allowing S4 to slide up and down in response to voltage changes. (**C**) The central pore of Na_v_1.5 channel in a closed state surrounded by two of the VSDs. On the left, a wild-type VSD is shown. When the S4 moves through the gating pore formed by S1 to S3, the positive charges on S4 interact with negative and aromatic amino acids (indicated by “−“). The VSD on the right has a substitution of the outermost arginine in S4 resulting in disruption of the interaction of the S4 and the gating pore. This results in an inward gating pore current, where mainly Na^+^ leaks in during closed state and depolarize the membrane potential.

Na_v_1.5 consists of a single protein with four homologue domains DI to DIV (Figure 2). Each of the domains contain six transmembrane segments, S1 to S6 (39, 40). S1 to S4 form the voltage sensing domain (VSD) of the protein. S5 and S6 from the four homologue domains form the central pore and the selectivity filter of the channel selectively allows passage of Na^+^ (39). The intracellular loop connecting DIII and DIV functions as an inactivation particle. Not surprisingly, LQTS3 variants are often found in this loop or its interaction sites on the cytoplasmic face of the channel. The S4 contains four to seven positive charges due to arginine or lysine residues. At resting membrane potentials, the positive residues are attracted to the negative cytosol. During depolarization of the membrane, the S4 segments are repelled causing a conformational change of the Na_v_1.5 protein resulting in opening of the activation gate of the central pore. The S1-S3 forms a canal or a “gating pore” that allows S4 to move in response to changes in membrane potential (41, 42). During gating, the S4 moves outward and rotates, while the positive charges on the S4 form ion pairs sequentially with highly conserved negative charged amino acids (aspartate and glutamate) and aromatic amino acids (tryptophan, phenylalanine and tyrosine) in S1, S2 and S3 (29, 41). This ion pairing neutralizes the positive charges on S4 during its transition through the gating pore. Remarkably, genetic variants that result in a substitution of either the positive charges in the S4 or the negative charges in S1–S3, can result in a leak current through the gating pore of the VSD, resulting in a “gating pore current” (29, 41) as illustrated in Figure 2C. Notice that the direction of the gating pore current depends on the position of the substitution (Figure 2C) and that gating pore currents can occur in either the closed or the open state of the channel (29). The gating pores are typically permeable for cations and the charge carrier as well as the direction of the current depends on the electrochemical driving force (29). Variants located in the inner region of S4 induce gating pore current in the open state (42) and the gating pore current is typically carried by Na^+^ and K^+^, which may shorten action potential duration. Variants in the outer region of S4 may cause a gating pore current in the closed state and the current is typically carried by Na^+^ due to electrochemical driving forces (17).

There is a clear predilection for Na_v_1.5 variants associated with MEPPC and DCM to localize to the VSD and in particular to the S4 as illustrated in Figure 1 (8, 11, 12, 29). The majority of the variants result in gain-of-function of Na_v_1.5, due to a shift in the voltage dependence of activation causing the channel to open at more negative voltages and/or increased “window current”, which represents a steady state current due to overlap of activation and inactivation properties of Na_v_1.5 (1, 12). However, other variants such as R225W (17, 32) and R1626H (37, 38) that cause a loss-of-function of Na_v_1.5 have also been associated with complex arrhythmias and DCM. This led Moreau and coworkers to suggest that the common modality for the *SCN5A* variants associated with DCM is the introduction of a gating pore current in Na_v_1.5 (29, 43)*.* As described above, gating pores are caused by disruption of interaction of the positive residues in the S4 with negative residues in S1–3 in the VSD, allowing ions to leak through the voltage sensitive domain. In support of this hypothesis, gating pore currents have been demonstrated for several S4 substitutions: R222Q (17), R225W (17), R225P (21), and R814W (21) have outward gating pore currents carried by Na^+^ and K^+^ at depolarized potentials where the channel is in an open state. Intriguingly, most of these variants also have increased window currents, which is a trademark of LQTS3 (1). Yet, the patients with MEPPC variants tend to have corrected QT (QTc) durations within the normal range (Table 1), suggesting that the increased window current is off-set by outward gating pore currents that will tend to shorten the QTc. For R222Q and R225W it has been demonstrated that prolonged depolarization causes the S4 to freeze in the open configuration resulting in inward gating pore currents carried by Na^+^ at negative potentials (17). This may explain the unstable resting membrane potential and hyper excitability that have been found in R222Q murine Purkinje fibers (14). For the G213D variant, Calloe and coworkers have recently reported inward gating pore current at negative potentials causing an unstable and depolarized resting membrane potential in cardiomyocytes derived from human induced pluripotent stem cells (13).

Most gating pore allows passage of different cations (29). Interestingly, the R219H variant that has been found in patients with PVCs and DCM (30, 31) has a H^+^ selective inward gating pore current at negative potentials (30). This proton gating pore may arise when an arginine is replaced by a histidine in the S4 thereby creating a proton wire (30, 31, 44). The R1626H variant has been found in patients with LQTS3 (38), lone AF (37), as well as ventricular tachycardia and DCM (own observation). The position of R1626H in the outer part of the S4 segment in DIV suggests it could induce a proton gating pore current at negative potentials similarly to R219H. Electrophysiological studies revealed a reduction in peak currents, a marked slowing of the onset of inaction and the presence of a late current (37), but the presence of gating pore currents remains to be tested. It should also be noted that the R1626H variant has been seen in the gnomAD allele frequency database at a significant number of individuals, see Table 1.

Even though the majority of DCM and MEPPC variants are found within the VSD, there are several variants found outside the VSD (Figure 2). It is conceivable that some of these regions interact with the VSD and may affect conformation changes or the electrical neutralization of the S4 during transition through the gating pore. Interestingly Barake and coworkers found that a third of LQTS3 patients also exhibited fascicular PVCs and Purkinje fiber hyper-excitability, which led them to suggest that the present of a persistent Na^+^ current due to a large window current can result in an MEPPC-like phenotype overlapping with LQTS3 (25). This suggest that increased persistent Na^+^ current, either through a gating pore or due to increased window currents could be the common modality in MEPPC.

It should be noted that gating pore currents are often undetected in routine patch clamp experiments as the gating pore currents are very small and resembles leak currents. It is critical that protocols optimized to detect gating pore currents are applied in functional characterization of *SCN5A* variants associated with complex arrhythmias and DCM.

## MEPPC and DCM

Whereas most genetic variants associated with hereditary DCM cause a change in structural genes (45), it is a subject of debate whether *SCN5A* is truly a DCM gene or the DCM is secondary to the arrhythmic burden. Peters and coworkers concluded that in all patients genotype-positive for an *SCN5A* variant, the DCM was secondary to the arrhythmic burden and no cases of DCM in the absence of arrhythmias were found, supporting that the arrhythmias are the defining feature of *SCN5A* variants associated with DCM (12).

Baman and coworkers found that a 24% PVC burden best separated the patient population with impaired left ventricular ejection fraction from patients with a preserved ejection fraction but a PVC burden as low as 10% may cause a reversible cardiomyopathy (46–48). This implicates that the PVCs are responsible for the MEPPC-associated DCM, even though the correlation between PVC burden and reduced LVEF is not linear and males appear to be at higher risk than females (3, 5, 6, 14).

The MEPPC-associated *SCN5A* variants typically result in a gain-of-function of Na_v_1.5 and a shift of the voltage dependence of activation making the cells more excitable (5, 13, 15). Increased sodium current either through the central pore or through a gating pore may directly depolarize and destabilize the resting membrane potential as well as lead to a cellular Na^+^ overload, which can activate the sodium calcium exchanger in reverse-mode, cause early after depolarization (14) and cellular Ca^2+^ overload (4, 8, 29). Ca^2+^ overload decreases Ca^2+^ sensitivity of the sarcomeres (49, 50) which may contribute to the development of cardiomyopathy. The Na^+^ overload may also predisposes to osmotic swelling and mitochondrial dysfunction (51) as well as activation of the sodium proton exchanger leading to acidification and uncoupling of gap junctions (52). Further, the premature contractions may lead to activation of baroreceptors and thereby increase sympathetic activity and cause myocardial stress (53).

## Clinical management

Current guidelines recommend catheter ablation in patients with DCM suspected to be caused by frequent and predominately monomorphic PVCs (54). However, ablation therapy has limited efficacy in eradiating MEPPC-associated PVCs (5, 9) as the whole Purkinje system is involved in triggering the ectopic contractions (5). Treatment with standard heart failure medical therapy including betablockers, ACE inhibitors and diuretics also has modest or no effect on MEPPC-associated DCM (3).

Several studies have reported that class 1c antiarrhythmic drugs, most notably flecainide (7, 13, 55) and quinidine are highly efficacious (5, 24). Flecainide and quinidine suppress PVCs and in longitudinal studies of the effect of flecainide treatment in MEPPC patients with the G213D (13) or R222Q (55) variant it was found that the reduction of the ectopic burden was associated with a reversibility of DCM. The reversible nature of DCM does not indicate that it is a benign cardiomyopathy (12). If not properly managed, the patients risk severe heart failure and sudden cardiac death (5, 12, 24). The presence of DCM in MEPPC patients is also a risk factor for malignant ventricular arrhythmias in itself (12), which underscores the importance of proper treatment and genetic testing including the *SCN5A* gene should be considered in young patients with a high PVC burden of multifocal origin and/or left bundle branch block morphologies.

Laurent and coworkers reported that cardiac resynchronization therapy (CRT) and amiodarone introduced simultaneously led to a partial normalization of left ventricular function in patients with the R222Q variant (5) but experience with CRT in MEPPC patients with left bundle branch block and heart failure is sparse. One report showed that atrial pacing at 90 beats per minute in combination with mexiletine in a patient carrying the R814W variant was effective in PVC suppression (24). Thus, it is possible that atrial overdrive pacing may also be an effective treatment strategy in patients with a high PVC burden despite medical antiarrhythmic treatment.

Given the rarity of the disease there is a need to establish a registry of *SCN5A* variants associated with MEPPC and DCM, in order to improve knowledge on clinical symptoms and pharmaceutical management.

## Conclusion

*SCN5A* genetic variants are associated with several cardiac arrhythmia syndromes including MEPPC as well as overlap syndromes. MEPPC is a relatively novel and ultrarare disease entity characterized by multifocal premature complexes originating from the Purkinje system and associated DCM. Increased Na^+^ current, either through a gating pore or due to increased window currents could be the common modality in MEPPC. MEPPC is important to diagnose as treatment with class 1c agents prevent morbidity and mortality, whereas standard treatment of PVCs and DCM with catheter ablation and heart failure medication is ineffective. Failure to detect MEPPC often results in inappropriate managing with potentially severe consequences for the patient and relatives.
